# Clinical features of *De Novo* acute myeloid leukemia with concurrent *DNMT3A*, *FLT3* and *NPM1* mutations

**DOI:** 10.1186/s13045-014-0074-4

**Published:** 2014-10-04

**Authors:** Sanam Loghavi, Zhuang Zuo, Farhad Ravandi, Hagop M Kantarjian, Carlos Bueso-Ramos, Liping Zhang, Rajesh R Singh, Keyur P Patel, L Jeffrey Medeiros, Francesco Stingo, Mark Routbort, Jorge Cortes, Rajyalakshmi Luthra, Joseph D Khoury

**Affiliations:** From the Department of Hematopathology, The University of Texas M.D. Anderson Cancer Center, 1515 Holcombe Boulevard, Unit 0072, Houston, TX 77030 USA; Department of Biostatistics, The University of Texas M.D. Anderson Cancer Center, 1515 Holcombe Boulevard, Unit 1411, Houston, TX 77030 USA; Department of Leukemia, The University of Texas M.D. Anderson Cancer Center, 1515 Holcombe Boulevard, Unit 0428, Houston, TX 77030 USA

**Keywords:** Acute myeloid leukemia, Next-generation sequencing, *DNMT3A*, *FLT3*, *NPM1*

## Abstract

**Background:**

*De novo* acute myeloid leukemia (AML) with concurrent *DNMT3A, FLT3* and *NPM1* mutations (AML^*DNMT3A/FLT3/NPM1*^) has been suggested to represent a unique AML subset on the basis of integrative genomic analysis, but the clinical features of such patients have not been characterized systematically*.*

**Methods:**

We assessed the features of patients (n = 178) harboring mutations in *DNMT3A, FLT3* and/or *NPM1,* including an index group of AML^*DNMT3A/FLT3/NPM1*^ patients.

**Results:**

Patients with AML^*DNMT3A/FLT3/NPM1*^ (n = 35) were significantly younger (median, 56.0 vs. 62.0 years; p = 0.025), mostly women (65.7% vs. 46.9%; p = 0.045), and presented with a higher percentage of bone marrow blasts (p < 0.001) and normal cytogenetics (p = 0.024) in comparison to patients within other mutation groups in this study. Among patients <60 years old, those with AML^*DNMT3A/FLT3/NPM1*^ had a shorter event-free survival (EFS) (p = 0.047). *DNMT3A* mutations and not *FLT3* or *NPM1* mutations were independently associated with overall survival (OS) (p = 0.026). Within mutation subgroups, patients with AML^*DNMT3A/NPM1*^ had a significantly shorter OS compared to those with AML^*FLT3-ITD/NPM1*^ (p = 0.047) suggesting that the adverse impact of *DNMT3A* mutations is more pronounced than that of *FLT3-*ITD among patients with *NPM1* mutation.

**Conclusions:**

*DNMT3A* has a significant dominant effect on the clinical features and outcomes of *de novo* AML patients with concurrent *DNMT3A, FLT3* and *NPM1* mutations.

**Electronic supplementary material:**

The online version of this article (doi:10.1186/s13045-014-0074-4) contains supplementary material, which is available to authorized users.

## Background

*DNMT3A, FLT3,* and *NPM1* mutations are among the most common genomic alterations in *de novo* acute myeloid leukemia (AML) and play a key role in the pathogenesis and evolution of the disease, particularly in the absence of AML-associated recurrent cytogenetic abnormalities [1,2]. *DNMT3A* encodes a DNA methyltransferase that catalyzes the addition of a methyl group to cytosine residues in CpG islands resulting in reduced expression of downstream genes. *DNMT3A* mutations, most commonly *DNMT3A*^*R882*^, are significantly enriched in patients with intermediate risk cytogenetics [1-4]. Mutations resulting in constitutive FLT3 activation include internal tandem duplication (*FLT3*-ITD) mutations and tyrosine kinase domain (*FLT3*-TKD) point mutations; *FLT3*-ITD in particular is a known adverse prognostic indicator in AML patients [2,5-13]. *NPM1* mutations are generally associated with more favorable outcomes in cytogenetically normal (CN)-AML in the absence of *FLT3*-ITD [6,14].

Integrative genomic analysis of *de novo* AML by The Cancer Genome Atlas (TCGA) consortium recently identified a subset of AML patients in which *DNMT3A*, *FLT3*, and *NPM1* mutations coexist at a higher frequency than would be expected for a chance occurrence [1]. Importantly, while this AML subset (herein referred to as AML^*DNMT3A/FLT3/NPM1*^) seems to have unique characteristics at the mRNA, miRNA, and epigenetic levels [1], its clinical features are not known. However, whereas the clinical implications of *FLT3* and *NPM1* mutations in *de novo* AML are largely well established, the impact of *DNMT3A* mutations on *NPM1* and/or *FLT3* mutations remains poorly understood especially in patients with AML^*DNMT3A/FLT3/NPM1*^ [15,16]. Several studies have suggested that the presence of *DNMT3A* mutations in AML is associated with poor clinical outcomes [3,4,17,18]. In one study, patients with AML harboring both *DNMT3A* and *FLT3*-ITD mutations relapsed more often than patients with either *DNMT3A* or *FLT3*-ITD alone [19]. Yet, in other studies *DNMT3A* mutations were not independently predictive of clinical outcomes in the overall AML population or specific subgroups [2,15,19-21].

In this study, we assess the clinical and outcome characteristics of patients with AML^*DNMT3A/FLT3/NPM1*^ as an index group and compare them to those with *de novo* AML harboring other mutation combinations involving *DNMT3A*, *FLT3* and/or *NPM1* to further elucidate the clinical effects of these mutations when they occur concurrently.

## Results

### Study group characteristics

**Figure 1:**
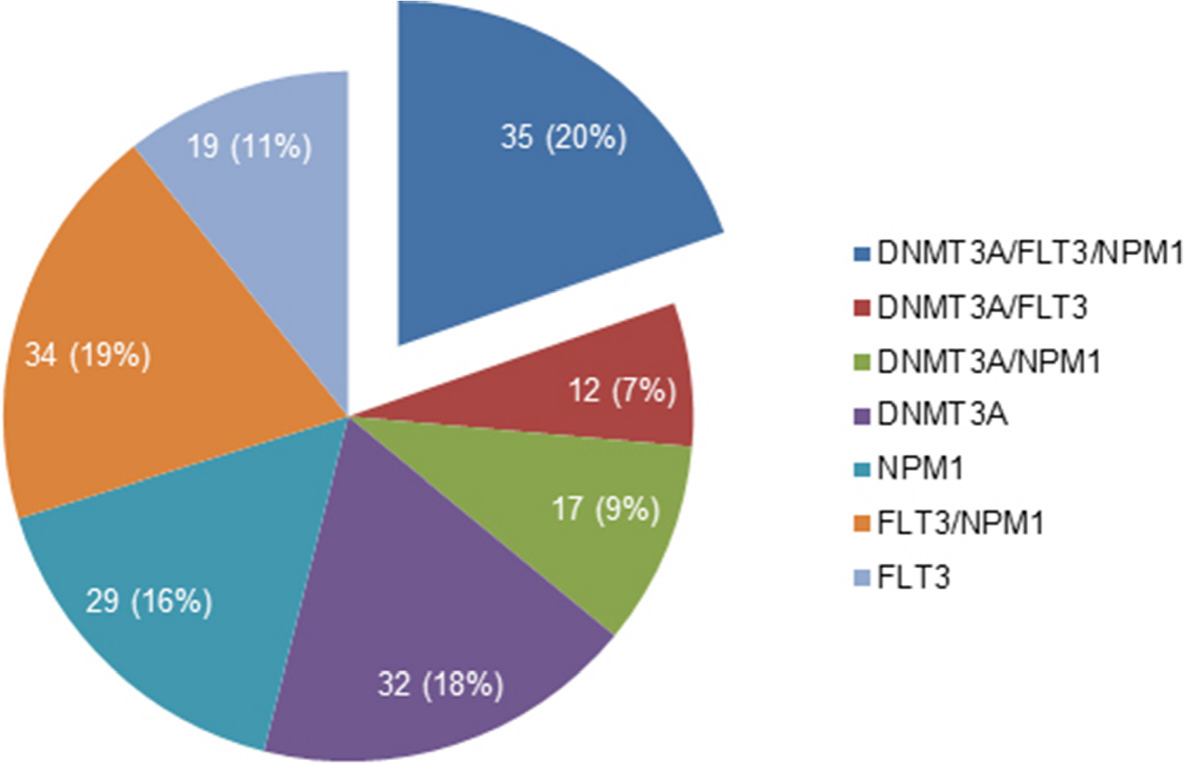
**Mutation subgroups of** ***de novo*** **acute myeloid leukemia included in the study.**

The study group consisted of 178 *de novo* AML patients with one, two, or three mutations involving the *DNMT3A, FLT3* and/or *NPM1* genes. The composition of the mutation subgroups is illustrated in Figure 1. The MD Anderson Cancer Center (MDACC) group (n = 85) consisted of 43 women and 42 men with a median age of 64 years (range, 23 to 91 years). They included 18 patients with AML^*DNMT3A/FLT3/NPM1*^ and 67 patients with the following mutations: *FLT3* and *NPM1* (AML^*FLT3/NPM1*^, n = 22); *NPM1* only (AML^*NPM1*^, n = 15)*; DNMT3A* only (AML^*DNMT3A*^, n = 13); *DNMT3A* and *FLT3* (AML^*DNMT3A/FLT3*^, n = 8); *DNMT3A* and *NPM1* (AML^*DNMT3A/NPM1*^, n = 7); and, *FLT3* only (AML^*FLT3*^, n = 2). The TCGA group (n = 93) consisted of 47 women and 46 men with a median age of 58 years (range, 21 to 83 years). They included 17 patients with AML^*DNMT3A/FLT3/NPM1*^ and 76 patients with the following mutations: *FLT3* and *NPM1* (AML^*FLT3/NPM1*^, n = 12); *NPM1* only (AML^*NPM1*^, n = 14)*; DNMT3A* only (AML^*DNMT3A*^, n = 19); *DNMT3A* and *FLT3* (AML^*DNMT3A/FLT3*^, n = 4); *DNMT3A* and *NPM1* (AML^*DNMT3A/NPM1*^, n = 10); and, *FLT3* only (AML^*FLT3*^, n = 17).

### Characteristics of AML^*DNMT3A/FLT3/NPM1*^

The characteristics of patients within mutation subgroups are summarized in Table 1. Since patients with concomitant mutations in *DNMT3A, FLT3* and *NPM1* (index group) had been reported to possibly represent a distinctive genomic subset of *de novo* AML with unique characteristics at the mRNA, miRNA, and epigenetic levels [1], their features were first compared to those of patients in other mutation groups. Patients with AML^*DNMT3A/FLT3/NPM1*^ were significantly younger (median 56.0 vs. 62.0 years; p = 0.025). Most were women (65.7% vs. 46.9%; p = 0.045) who presented with a heavy disease burden, namely an elevated WBC (50.9 vs. 19.0 × 10^3^/μL; p = 0.006) and BM blast counts (83.5% vs. 71.0%, p < 0.001).

**Table 1:** **Demographic, clinical and laboratory characteristics of patients in the study group**

**Variable**		**AML** ^***DNMT3A/FLT3/NPM1***^	Other*	**P-value**
**Age** (years)	Median	56.00	62.00	**0.025**
	Range	29-81	21-91	
**Sex**	Female (%)	23 (65.7)	67 (46.9)	**0.045**
	Male (%)	12 (34.3)	76 (53.1)	
**BM Blast %** (range)	Median	83.5 (29-98)	71.0 (20-99)	**<0.001**
Hemoglobin g/dL (range)	Median	9.6 (6-13)	9.3 (6-14.3)	0.683
**WBC** 10^3^/μL (range)	Median	50.9 (0.1-298)	19.0 (0.3-297)	**0.006**
Monocytes 10^3^/μL (range)	Median	4.0 (0-76)	5.0 (0-73)	0.960
Platelets 10^3^/μL (range)	Median	45.0 (7-218)	50.0 (8-1069)	0.397
PB Blast % (range)	Median	46.0 (0-99)	23.0 (0-99)	0.191
CG Risk Group %^†^	Intermediate	91.4	80.4	0.168
	Poor	2.9	14.0	
**CN-AML%**		87.5	67.4	**0.024**

*Includes: All mutation groups other than AML^*DNMT3A/FLT3/NPM1*^.

*Abbreviations*: *AML* acute myeloid leukemia, *BM* bone marrow, *PB* peripheral blood, *WBC* white blood cell count, *CG* cytogenetic, *CN-AML* cytogenetically normal AML.

Bold font indicates parameters with significant differences between groups.

Morphologically, AML^*DNMT3A/FLT3/NPM1*^ tended to be associated with myelomonocytic blast morphology, which corresponds to the M4 and M5 categories in the French American British (FAB) classification [22]. Among 32 AML^*DNMT3A/FLT3/NPM1*^ cases with FAB classification data, 10 (31%) were M4; 8 (25%) were M5; 6 (19%) were M1; 6 (19%) were M2; 1 (3%) was M0; and, 1 (3%) was refractory anemia with excess blasts in transformation. None of these cases displayed sufficient dysplasia in background hematopoietic elements to warrant classification as AML with myelodysplasia-related changes. In most cases blasts showed “cup-like” nuclear morphology, which had been described previously in association with *FLT3-ITD* and *NPM1* mutations [23,24]. Namely, blasts with cup-like nuclear morphology were identified in 17/18 (94.4%) AML^*DNMT3A/FLT3/NPM1*^ cases and comprised a variable subset of total blasts, ranging from 3-53% (median 9%; mean 16.1%). In 10/18 (55.5%) cases, such blasts comprised >10% of total blasts.

By flow cytometry immunophenotyping (MDACC group), all AML^*DNMT3A/FLT3/NPM1*^ had a definite myeloid immunophenotype. In all cases, blasts were positive for CD13, CD33, and CD123 and negative for surface and cytoplasmic CD3 expression. Myeloperoxidase expression was detected in 11/14 (78.6%) cases. Blasts were positive for CD34 in 13/18 (72.2%) cases. In accordance with myelomonocytic morphology, blasts expressed one or more monocyte-associated antigens (CD4, CD14, CD64) in 11/18 (61.1%) cases. In one case, myeloid blasts co-expressed the B-cell marker CD19. (Additional file 1: Figure S1).

A significant association was observed between AML^*DNMT3A/FLT3/NPM1*^ and diploid karyotype (CN-AML) (p = 0.024). Among 33 AML^*DNMT3A/FLT3/NPM1*^ patients with available cytogenetics data, 32 (97%) had intermediate-risk cytogenetics and included 28 (85%) CN-AML. Only 1 (3%) patient had poor-risk cytogenetics. Notably, none of the patients had recurrent AML-associated cytogenetic abnormalities.

**Figure 2:**
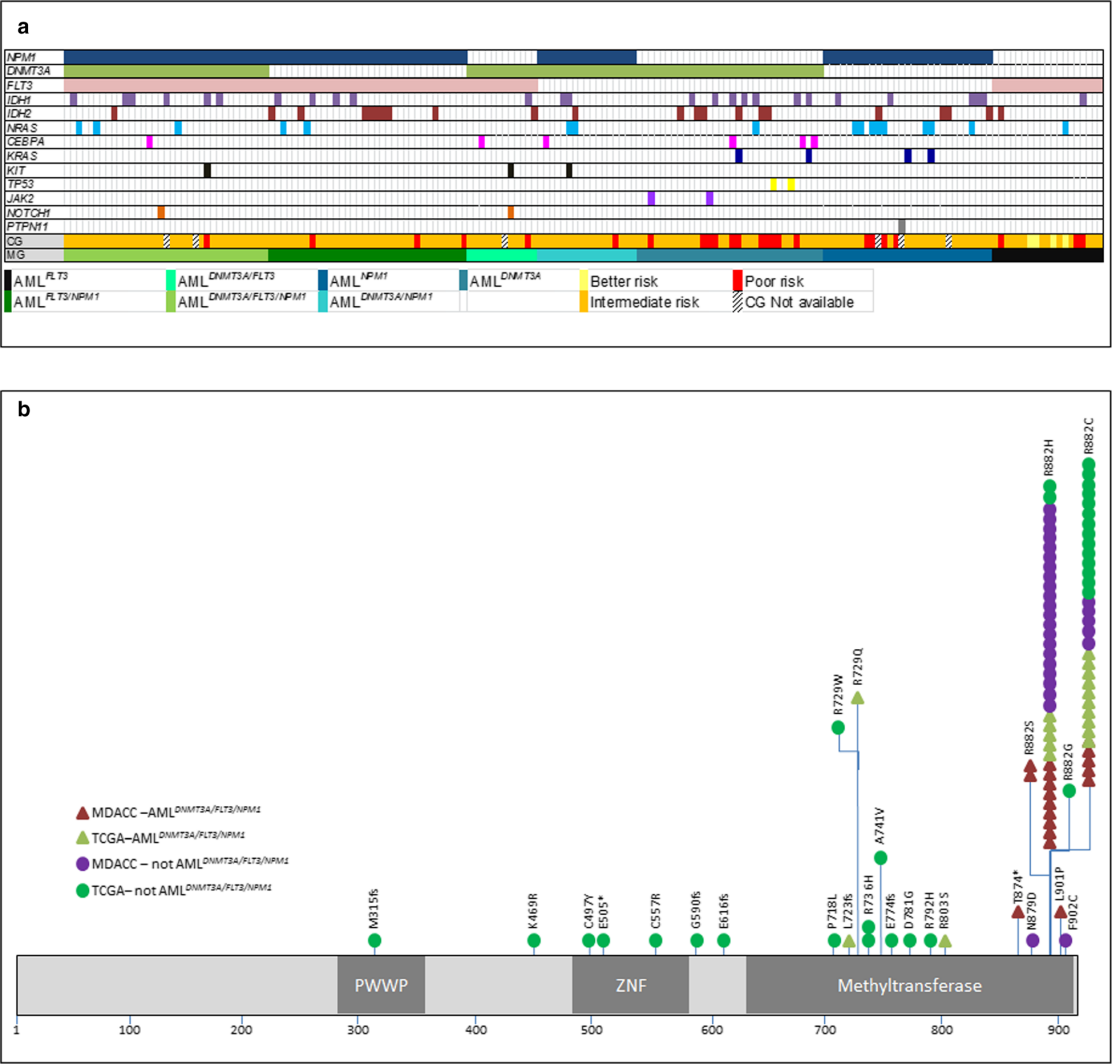
**Schematic representation of mutations. (a)** Mutation status of genes assessed in all patients in the study group. Each column represents an individual case. The bottom rows represent, respectively, the cytogenetic risk group (CG) and mutation group (MG) per corresponding color designation. Assessed genes in which mutations were not detected are not included in this diagram. Mutation detection techniques in the MDACC and TCGA groups were different (see Methods section). **(b)** Distribution of genomic variants within *DNMT3A* detected in the study group. PWWP represents a region encoding for a highly conserved proline-tryptophan-tryptophan-proline motif. ZNF represents the ADD (*ATRX, DNMT3*, and *DNMT3L*)-type zinc finger domain.

A summary of mutations in genes assessed in both the MDACC and TCGA groups is provided in Figure 2a. All *DNMT3A* variants detected in patients with AML^*DNMT3A/FLT3/NPM1*^ were missense substitutions in exon 23, and most involved codon 882 resulting in the substitution of arginine by histidine (14/35; 40%) or cysteine (14/35; 40%). Mutations in other codons - 882 (arginine > serine), 901 (leucine > proline), 874 (tyrosine-stop), 803 (arginine to serine), 855 (lysine to threonine) and 723 (frameshift) *-* were detected with less frequency. (Figure 2b) (Additional file 2: Table S1).

*FLT3* mutations in the AML^*DNMT3A/FLT3/NPM1*^ group included *FLT3*-ITD in 24/35 (69%), *FLT3*-TKD^D835^ in 9/35 (26%), and simultaneous *FLT3*-ITD/TKD^D835^ mutations in 2/35 (6%). There was no significant difference in *FLT3* mutation types between the AML^*DNMT3A/FLT3/NPM1*^ and the other mutations groups (p = 0.108) (Additional file 2: Table S2). *NPM1* mutations uniformly involved exon 12 in all MDACC cases. Other mutations observed in AML^*DNMT3A/FLT3/NPM1*^ included *IDH1* (17.1%), *NRAS (8.5%*), *KIT* (2.8%), *IDH2* (2.8%), *CEBPA* (2.8%) and *NOTCH1* (2.8%). None of the cases had mutations in *TP53.*

**Figure 3:**
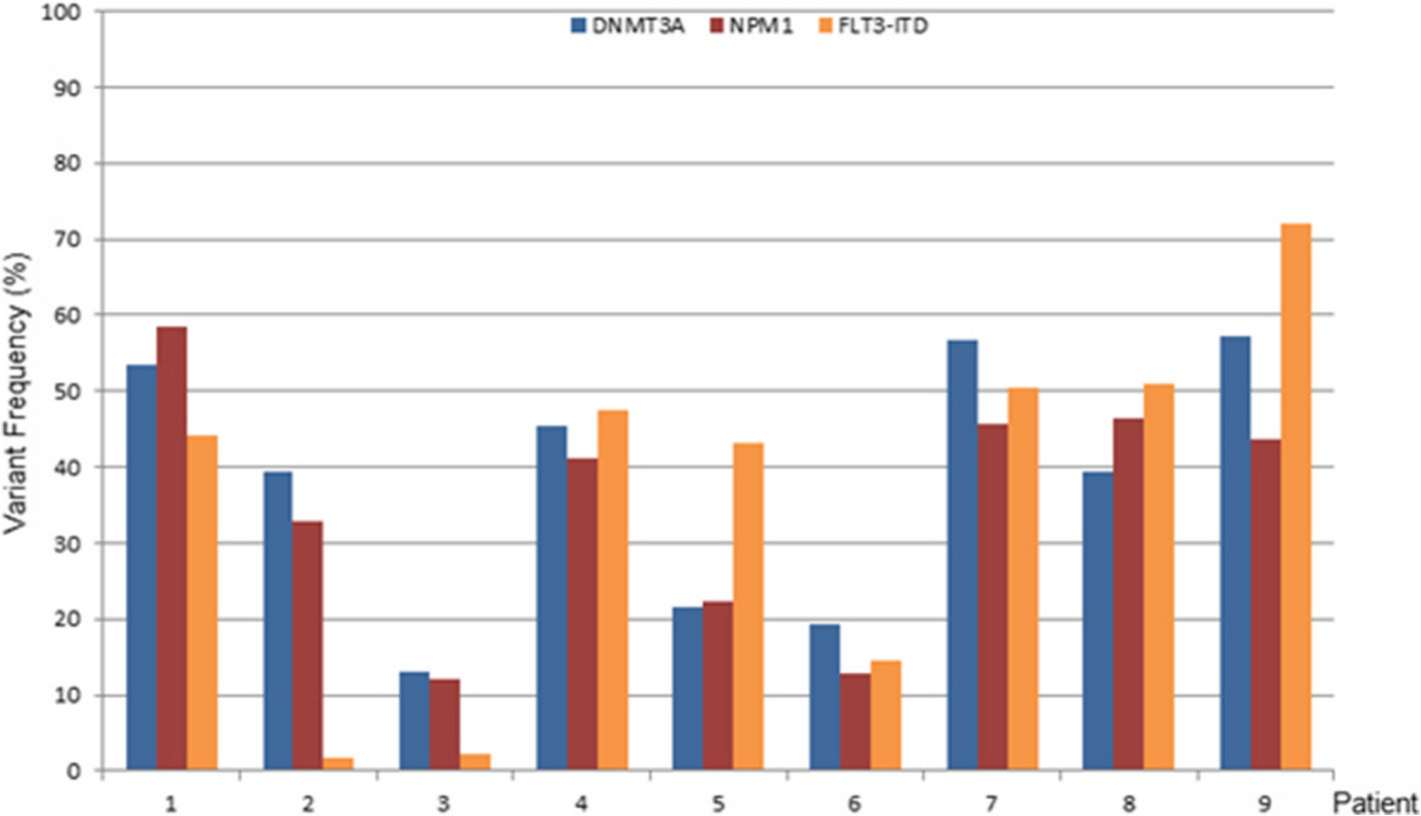
**Estimated** ***DNMT3A, FLT3*** **, and** ***NPM1*** **variant (allelic) frequencies in a subset of** ***de novo*** **acute myeloid leukemia samples harboring all three mutations.**

To assess the relative dominance of mutations in individual cases within the AML^*DNMT3A/FLT3/NPM1*^ group, we estimated the variant frequency of each of the mutant *DNMT3A, FLT3*, and *NPM1* alleles in a subset MDACC samples and found them to be generally commensurate, suggesting that they commonly co-occur within a dominant clone (Figure 3). In addition, the estimated variant frequency suggested that these mutations are heterozygous.

### Prognostic impact of concurrent *DNMT3A*, *FLT3*, and *NPM1* mutations

Follow-up data was available for 84/85 patients in the MDACC group with a median follow up of 11.8 months (range, 0.6-69.0 months). Of these patients, 50/84 presented to our institution at diagnosis prior to induction chemotherapy (4/50 with AML^*DNMT3A/FLT3/NPM1*^), 9 presented with primary refractory disease (2/9 with AML^*DNMT3A/FLT3/NPM1*^), and 20 presented in relapse (10/20 with AML^*DNMT3A/FLT3/NPM1*^). An additional 5 patients (2/5 with AML^*DNMT3A/FLT3/NPM1*^) were in remission at the time of referral to our institution. Allogeneic SCT therapy was administered to 25 patients (7/25 with AML^*DNMT3A/FLT3/NPM1*^) in the MDACC group. When the actual last follow up date was considered, no difference in OS was observed between those who received allo-SCT and those who did not (p = 0.203); similarly, no difference in OS was observed between AML^*DNMT3A/FLT3/NPM1*^ patients who received allo-SCT and those who did not (p = 0.186). At last follow up, 32 MDACC patients (11 AML^*DNMT3A/FLT3/NPM1*^) were dead of disease. Importantly, there was no identifiable bias in survival outcomes on the basis of therapy selection (high-intensity vs. low-intensity, and “3 + 7” vs. “non-3 + 7”) for any of the prognostic parameters assessed by univariate or multivariate modeling in the MDACC group. Survival data was available for 87/93 patients in the TCGA group with a median follow up of 9.2 months (range, 0.9-62.8 months). At last follow up, 60 patients (13 AML^*DNMT3A/FLT3/NPM1*^) in the TCGA group were dead of disease, the majority during first relapse.

**Figure 4:**
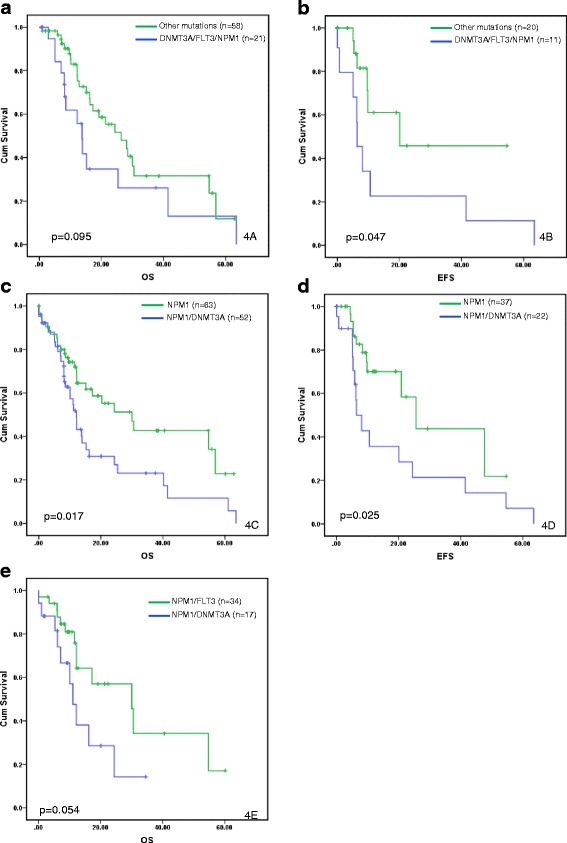
**Survival analysis by mutation status.** Overall survival **(a)** and event-free survival **(b)** of *de novo* acute myeloid leukemia patients younger than 60 years of age with concomitant mutations in *DNMT3A, FLT3*, and *NPM1* (AML^*DNMT3A/FLT3/NPM1*^) in comparison to those within other mutation subgroups in this study. Overall survival **(c)** and event-free survival **(d)** of patients with AML harboring *NPM1* mutation in conjunction with wild-type *DNMT3A* (NPM1 group) or mutant *DNMT3A* (NPM1/DNMT3A group). Overall survival **(e)** of patients with CN-AML harboring *NPM1* mutations with concomitant *FLT3* (NPM1/FLT3 group) or *DNMT3A* (NPM1/DNMT3A group).

Younger (<60 years) patients with AML^*DNMT3A/FLT3/NPM1*^ had a significantly shorter EFS (p = 0.047) and a tendency towards shorter OS (p = 0.095) compared to those in the other mutation groups (Figure 4a-b). Although patients with AML^*DNMT3A/FLT3/NPM1*^ tended to have shorter OS compared to those in the other mutation groups, the difference was not significant in the entire group as well as within the CN-AML group (p = 0.211 and p = 0.209, respectively). Importantly, there was no significant difference in OS among AML^*DNMT3A/FLT3/NPM1*^ patients with *FLT3-ITD* and *FLT3-TKD* mutations types (p = 0.822).

To begin understanding the differential clinical impact of *DNMT3A* mutations relative to *FLT3* and *NPM1* on outcomes in *de novo* AML, including AML^*DNMT3A/FLT3/NPM1*^, we then turned our attention to multivariate analysis. The latter demonstrated that age (p = 0.019), *DNMT3A* mutations (p = 0.026), *IDH1* mutation (p = 0.002), and cytogenetics (poor-risk vs. intermediate/better-risk) (p = 0.018) were independently associated with OS. Poor-risk cytogenetics followed by *DNMT3A* mutations were the strongest unfavorable factors, while *IDH1* mutation was favorable (Table 2). The adverse effect of *DNMT3A* mutations (p = 0.039) persisted when only younger (<60 years) patients were considered in multivariate analysis. To control for the impact of cytogenetics, we proceeded to assess OS and EFS as a function of *DNMT3A* mutation status among patients with CN-AML. Indeed, *DNMT3A* mutations within the CN-AML subset were associated with significantly shorter OS and EFS (p = 0.044 and p = 0.051, respectively). (Additional file 3: Figure S2A-B) The adverse impact of *DNMT3A* mutations on OS was particularly notable among CN-AML patients <60 years of age (p = 0.014). These data suggest that in the absence of high-risk cytogenetics, *DNMT3A* mutations exert an independent impact on *de novo* AML outcomes including those of the AML^*DNMT3A/FLT3/NPM1*^ group.

**Table 2:** **Multivariate analysis of the impact of demographic, laboratory, and molecular parameters on overall survival in the entire study group**

	**P-value**	**HR**	**95% CI**	
			**Lower**	**Upper**
**Age**	**0.019**	1.032	1.005	1.060
Sex	0.688	0.881	0.476	1.631
*FLT3* mutation	0.222	1.521	0.775	2.985
NPM1 mutation	0.476	1.306	0.627	2.720
***DNMT3A*** **mutation**	**0.026**	2.552	1.120	5.816
***IDH*** **1 mutation**	**0.002**	0.223	0.085	0.588
*IDH* 2 mutation^†^	0.134	0.407	0.125	1.320
Poor risk cytogenetics*	**0.018**	4.584	1.297	16.199
CN-AML	0.358	1.596	0.589	4.328
BM blast percentage	0.487	0.995	0.981	1.009
PB blast percentage	0.692	1.002	0.992	1.012
Hemoglobin	0.864	0.984	0.816	1.186
White blood count	0.640	1.002	0.995	1.008

^†^Include 17 cases with *IDH2* ^*R140*^ and 2 with *IDH2* ^*R172*^ [25].

*Poor-risk vs intermediate and better risk categories per National Comprehensive Cancer Network guidelines (version 2.2013) [26].

*Abbreviations*: *HR* hazards ratio, *CI* confidence interval, *BM* bone marrow, *PB* peripheral blood, *CN-AML* cytogenetically normal (diploid) acute myeloid leukemia.

Bold font indicates parameters with significant impact on overall survival.

We then asked whether the dominant effect of *DNMT3A* mutations seen in AML^*DNMT3A/FLT3/NPM1*^ is observed in AML with either *NPM1* or *FLT3* mutations, which are important factors in risk stratification particularly for younger patients with CN-AML [26,27]. Notably, among patients with *NPM1* mutation, those with CN-AML^*NPM1/DNMT3A*^ had significantly shorter OS and EFS compared to those with CN-AML^*NPM1*^ and wild-type *DNMT3A* (p = 0.022 and p = 0.040, respectively). These differences in OS and EFS were also observed within the entire AML group (p = 0.017 and p = 0.025, respectively) (Figure 4c-d). Importantly, the adverse impact of *DNMT3A* mutations on OS among AML^*NPM1*^ patients was particularly pronounced in those <60 years of age, within both the entire group and the CN-AML group (p = 0.008 and p = 0.014, respectively). Furthermore, patients with CN-AML^*DNMT3A/NPM1*^ had shorter OS and EFS compared to those with CN-AML^*FLT3-ITD/NPM1*^ (p = 0.047 and p = 0.072, respectively). This effect on OS and EFS persisted when all *FLT3-*mutated (ITD and TKD) cases were included (p = 0.054 and p = 0.051, respectively) (Figure 4e) and suggests that the impact of *DNMT3A* mutations is more pronounced than that of *FLT3* (ITD and TKD) in CN-AML^*NPM1*^. Conversely, among all patients with *DNMT3A*, there was no significant difference in OS or EFS between AML^*DNMT3A/NPM1*^ and AML^*DNMT3A/FLT3*^ (p = 0.301 and p = 0.989, respectively), suggesting that the dominant effect of *DNMT3A* mutations is not modulated by the presence of *NPM1* or *FLT3* mutations. Finally, when we compared OS and EFS for patients with AML^*DNMT3A/FLT3/NPM1*^ (n = 35) to that of patients with AML^*FLT3/NPM1*^ (n = 34) we noticed that the former group tended to have worse OS and EFS, although the difference was not statistically significant (Additional file 4: Figure S3).

## Discussion

Integrative genomic analysis data from TCGA suggest that AML^*DNMT3A/FLT3/NPM1*^ is a unique disease subset with distinctive features at the mRNA, miRNA, and epigenetic levels, but the clinical features and outcome characteristics of this AML subset remain unknown. By simultaneously harboring *DNMT3A, FLT3* and *NPM1* mutations, AML^*DNMT3A/FLT3/NPM1*^ offers a unique advantage to study the differential impact of these genes in AML. This is particularly important because although the clinical implications of *FLT3* and *NPM1* mutations in *de novo* AML have been well characterized, their prognostic impact in the presence of concurrent *DNMT3A* mutations has not been assessed systematically. Accordingly, we designed this study to investigate the clinical and outcome characteristics of patients with AML^*DNMT3A/FLT3/NPM1*^ as an index group and compare them to those with *de novo* AML harboring other mutation combinations involving *DNMT3A*, *FLT3* and/or *NPM1*. Since the incidence of AML^*DNMT3A/FLT3/NPM1*^ is low, cases seen at our institution were combined with those in the TCGA data set to increase the statistical power of our analysis. The inclusion of patients from two different cohorts and the combination of MDACC patients with untreated disease along with those who presented in relapse or with persistent leukemia for statistical purposes are acknowledged limitations of this retrospective study. Notwithstanding, our data demonstrate a significant association between AML^*DNMT3A/FLT3/NPM1*^ and the following clinical features: young age, female gender, diploid cytogenetics, poor EFS and OS particularly in patients <60 years of age, and a heavy disease burden manifested by elevated peripheral blood and bone marrow blast counts. These features of AML^*DNMT3A/FLT3/NPM1*^ are reminiscent of the salient clinical attributes described broadly for AML patients with *DNMT3A* mutations [1-4].

In the NCCN [26] and ELN [27] risk classification systems for AML, due importance is placed on *NPM1* and *FLT3* but not *DNMT3A* mutations in CN-AML. In our study group, patients with *DNMT3A* mutations had worse outcomes than those with wild-type *DNMT3A*. Hou *et al.* [28] and Ribeiro *et al.* [29] have similarly demonstrated that *DNMT3A* mutations are independent predictors of shorter OS in patients with *de novo* AML. Shirarov *et al.* [30] also demonstrated in their study that *DNMT3A* mutations were associated with worse outcome including significantly shorter OS and EFS and are an independent determinate of worse outcome in younger patients with CN-AML. In addition, recent data demonstrate that *DNMT3A* mutation status has an impact on therapy selection; in one study, patients with *DNMT3A* mutations who were treated with high-dose daunorubicin compared to standard-dose therapy were shown to have longer OS [2].

Our findings suggest that in the absence of high-risk cytogenetics, *DNMT3A* mutation status has an impact on outcome in the presence of *FLT3* and/or *NPM1*. In this study group, mutant *DNMT3A* is associated with adverse outcomes among CN-AML patients that have *NPM1* mutations. This effect appears more pronounced than that of either *FLT3*-ITD or *FLT3*-TKD mutations and is not mitigated by *NPM1* or dependent on the presence of cytogenetic abnormalities. However, by current risk stratification schemes CN-AML^*DNMT3A/NPM1*^ patients would be regarded as a better/favorable risk group whereas they likely should be reclassified in higher risk categories. Along similar lines, Kihara *et al* suggested recently that ELN risk stratification can be enhanced by reassigning patients in the Favorable risk category to the Intermediate-I group if they have *DNMT3A* mutations [31]. Data from this study suggests that among patients with *de novo* CN-AML, particularly those <60 years, AML^*DNMT3A/FLT3/NPM1*^ patients seem to have the worst clinical outcomes, followed by those with AML^*FLT3/DNMT3A*^ and then those with AML^*NPM1/DNMT3A*^.

## Methods

### Patient study group

This study was approved by the MDACC Institutional Review Board. We searched our records for patients with *de novo* AML and known mutation status for each of *DNMT3A*, *FLT3* and *NPM1* who presented to our institution between 3/2009 and 12/2013. All patients met the following study inclusion requirements: 1) positive for mutations in *DNMT3A*, *FLT3* and/or *NPM1*; 2) 18 years of age or older at diagnosis; 3) acute myeloid leukemia diagnosis as defined in the World Health Organization classification [32]. Patients with therapy-related AML, history of myelodysplastic syndrome or myeloproliferative neoplasms, and AML with t(15;17) were excluded.

The demographic, laboratory, and clinical data on patients in the study group were collected by chart review with emphasis on variables of demonstrated prognostic utility in AML [33]. Flow cytometry immunophenotyping and cytogenetic analyses were performed as part of routine clinical workup using previously described techniques [34,35]. Patients were allocated to intermediate- and high-risk cytogenetic risk groups per NCCN guidelines (version 2.2013) [26]. A detailed review of treatment history for all patients in the MDACC study group was undertaken, and patients were divided into high-intensity (cytarabine and anthracycline based induction regimens, further subdivided into “3 + 7” and “non-3 + 7”) and low-intensity therapy groups.

As reported previously [1], the TCGA cohort consisted of 200 adult patients with various subtypes of *de novo* AML. To increase the statistical power of our analysis and encompass a broader cohort of *de novo* AML patients, TCGA patients who had mutations in *DNMT3A*, *FLT3* and/or *NPM1* were included in our study; those with t(15;17) were excluded. Publicly available annotated exome and whole-genome sequencing data as well as associated patient clinical data files were downloaded from the TCGA Data Portal (https://tcga-data.nci.nih.gov/tcga/) at various time points between September 2013 and January 2014. Patients were allocated to cytogenetic risk groups as described above. Patients in the TCGA group were treated per NCCN guidelines [1]. Patient enrollment and utilization of data were conducted in accordance with TCGA Human Subjects Protection and Data Access Policies [36].

### Mutation analysis

For NGS-based mutation screening of MDACC cases, analysis of hotspot genomic areas was performed by paired-end sequencing using the MiSeq (Illumina, San Diego, CA) sequencer and the TruSeq Amplicon Cancer Panel kit (Illumina) with 5 additional customized probe pairs targeting *DNMT3A* (exon 23, codons 866-913)*, XPO1, KLHL6, MYD88* and *EZH2* as described previously [37]. Variant (allelic) frequency was estimated as the percentage of variant reads over total reads at a specific allelic location. Matched germline DNA was not available for comparison. *FLT3* (ITD and TKD) and *NPM1* mutations were also screened using polymerase chain reaction (PCR) followed by capillary electrophoresis on Genetic Analyzer (Applied Biosystems, Foster City, CA), as described previously [38]. NGS-based mutation analysis and testing for *FLT3* and *NPM1* status are routinely performed on all patients with new onset or relapsed acute leukemia at our institution irrespective of their cytogenetic risk groups. The TCGA mutation analysis data was derived from whole genome and exome NGS and analyzed alongside matched germline DNA [1].

### Statistical analysis

Categorical and continuous variables were analyzed using Pearson Chi-square and Kruskal-Wallis rank sum tests, respectively. Overall survival (OS) was estimated by the Kaplan-Meier method using as a reference time point the date of initial AML diagnosis until death from any cause or date of last follow-up. Event-free survival (EFS) represented the duration of disease between the initial diagnosis and first relapse. No EFS data was available for TCGA patients. For MDACC patients who received allogeneic hematopoietic stem cell transplant (allo-SCT), the date of transplant was assigned as the last follow-up date. Survival curves were compared by the log rank test. Differences between two groups were considered significant if p-values were <0.05 in a two-tailed test. Multivariate analysis was performed by Cox proportional regression model. Significant factors were identified by Wald backward stepwise elimination.

## Additional files

Additional file 1: Figure S1. Immunophenotypic features of AML with *DNMT3A, FLT3*, and *NPM1* mutations as assessed by flow cytometry (n = 18). Color legend: red = positive; nude = partial positive, blue = negative; blank = not assessed. Additional file 2: Table S1.
*DNMT3A* mutation types in the study group. **Table S2.** *FLT3* mutation types in the study group. Additional file 3: Figure S2. Overall survival and event-free survival of *de novo* acute myeloid leukemia patients with mutant *DNMT3A* compared to those with wild-type *DNMT3A.*
Additional file 4: Figure S3. Overall survival of *de novo* acute myeloid leukemia patients with *DNMT3A, FLT3*, and *NPM1* mutations compared to those with *FLT3* and *NPM1* mutations and wild-type *DNMT3A.*
