# Supplementary figures and images for: Clinical features of De Novo acute myeloid leukemia with concurrent DNMT3A, FLT3 and NPM1 mutations
Source: J Hematol Oncol. 2014 Oct 4;7:74. doi: 10.1186/s13045-014-0074-4 (PMC4197326; doi:10.1186/s13045-014-0074-4)

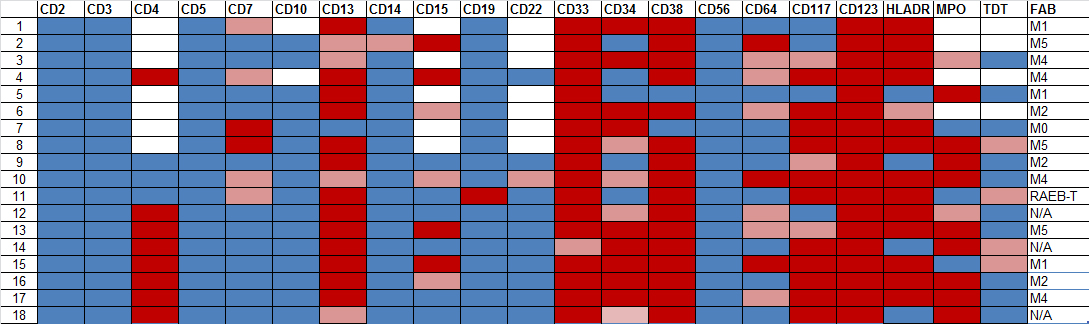

Supplement: Additional file 1: Figure S1. — Immunophenotypic features of AML with DNMT3A, FLT3, and NPM1 mutations as assessed by flow cytometry (n = 18). Color legend: red = positive; nude = partial positive, blue = negative; blank = not assessed. [file 13045_2014_74_MOESM1_ESM.jpeg]

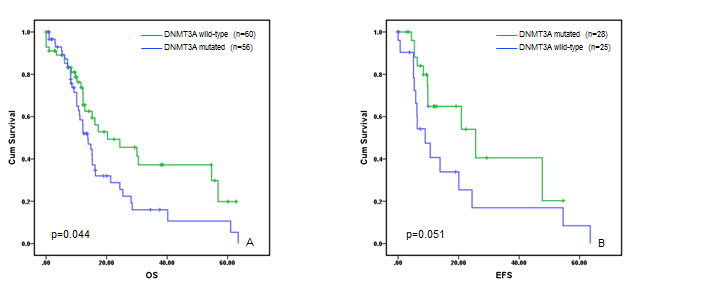

Supplement: Additional file 3: Figure S2. — Overall survival and event-free survival of de novo acute myeloid leukemia patients with mutant DNMT3A compared to those with wild-type DNMT3A. [file 13045_2014_74_MOESM3_ESM.jpeg]

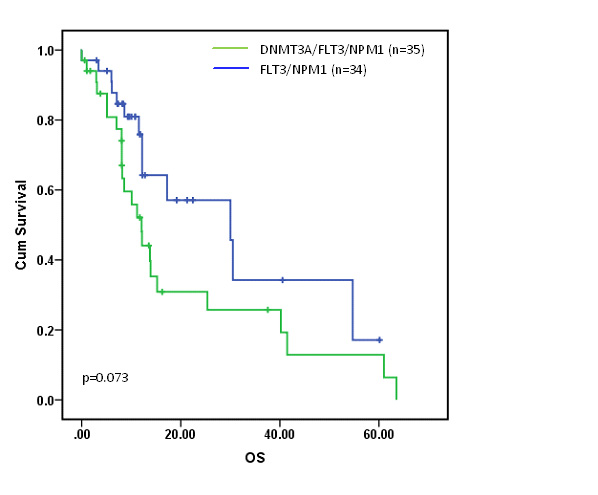

Supplement: Additional file 4: Figure S3. — Overall survival of de novo acute myeloid leukemia patients with DNMT3A, FLT3, and NPM1 mutations compared to those with FLT3 and NPM1 mutations and wild-type DNMT3A. [file 13045_2014_74_MOESM4_ESM.jpeg]
