# Supplementary material for: Clinical features of De Novo acute myeloid leukemia with concurrent DNMT3A, FLT3 and NPM1 mutations
Source: J Hematol Oncol. 2014 Oct 4;7:74. doi: 10.1186/s13045-014-0074-4 (PMC4197326; doi:10.1186/s13045-014-0074-4)
Supplement: Additional file 2: Table S1. — DNMT3A mutation types in the study group. Table S2. FLT3 mutation types in the study group. [file 13045_2014_74_MOESM2_ESM.docx]

**SUPPLEMENTAL MATERIAL**

**Supplemental Tables**

**Supplemental Table 1.** *DNMT3A* mutation types in the study group.

|  | Non-R882 | R882 | Total |
| --- | --- | --- | --- |
| AML*^DNMT3A/FLT3/NPM1^* | 4 (11.4%) | 31 (88.6%) | 35 |
| Others | 18 (29.5%) | 43 (70.5%) | 61 |
| Total | 22 | 74 | 96 |

Abbreviations: AML: acute myeloid leukemia; R: arginine.

**Supplemental Table 2.** *FLT3* mutation types in the study group.

|  | FLT3-ITD | FLT3-TKD | Both | Total |
| --- | --- | --- | --- | --- |
| AML*^DNMT3A/FLT3/NPM1^* | 24 (68.6%) | 9 (25.7%) | 2 (5.7%) | 35 |
| Others | 51 (78.4%) | 13 (20%) | 1 (5%) | 65 |
| Total | 75 | 22 | 3 | 100 |

Abbreviations: AML: acute myeloid leukemia; ITD: internal tandem duplication; TKD: tyrosine kinase domain mutation (D835).
